# Biological activity of the thyroid TRK-T3 oncogene requires signalling through Shc

**DOI:** 10.1038/sj.bjc.6600544

**Published:** 2002-09-04

**Authors:** E Roccato, C Miranda, V Ranzi, M Gishizki, M A Pierotti, A Greco

**Affiliations:** Department of Experimental Oncology, Istituto Nazionale Tumori, Via G. Venezian 1, 20133 Milan, Italy; SUGEN, 230 East Grand Ave, South San Francisco, California, CA 94080, USA

**Keywords:** TRK-T3, Shc, NTRK1, signal transduction, transforming activity

## Abstract

The thyroid TRK-T3 oncogene, produced by a chromosomal translocation, is a chimeric, constitutively activated version of the NTRK1/NGF receptor and it is able to transform NIH3T3 cells and differentiate PC12 cells. TRK-T3 oncoprotein triggers multiple signal transduction pathways. Among others, TRK-T3 binds and phosphorylates the Shc and SNT1/FRS2 adaptor proteins both involved in coupling the receptor tyrosine kinase to the mitogen-activated protein kinase pathway by recruiting Grb2/SOS. We were interested in defining the role of Shc in the oncogenesis by TRK-T3. The mutation of TRK-T3 tyrosine 291, docking site for both Shc and FRS2, abrogates the oncogene biological activity. To directly explore the role of Shc we used the ShcY317F mutant, which carries the mutation of a tyrosine residue involved in Grb2 recruitment. We demonstrated that the ShcY317F mutant exerts an inhibitory effect on TRK-T3 transforming activity. Such effect can be modulated by the amount of ShcY317F protein and affects the viability of cells expressing TRK-T3 by means of a mechanism involving apoptosis. Our results indicate a definitive role of the adaptor protein Shc in TRK-T3 transforming activity.

*British Journal of Cancer* (2002) **87**, 645–653. doi:10.1038/sj.bjc.6600544 www.bjcancer.com

© 2002 Cancer Research UK

## 

The thyroid TRK oncogenes are created by chromosomal rearrangements fusing the tyrosine kinase (TK) domain of the NTRK1/NGF receptor to the 5′-end portion of different activating genes (Pierotti *et al*, 1996). In the TRK-T3 oncogene, produced by a t(1;3) translocation, the NTRK1 TK domain is juxtaposed to sequences of the TFG gene (Greco *et al*, 1995), which encodes a protein of unknown function containing a coiled-coil domain, putative phosphorylation sites for PKC and CK2 and glycosylation sites (Mencinger *et al*, 1997; Mencinger and Aman, 1999). The TFG coiled-coil domain plays an important role in TRK-T3 activation and its consequent biological activity insofar as it mediates the dimerization leading to the ligand-independent tyrosine phosphorylation of the TK domain. Moreover, also the regions outside the coiled-coil domain are required for TRK-T3 transforming activity (Greco *et al*, 1998) in ways that are currently under investigation.

Signalling by the NTRK1 receptor has been extensively studied and it involves both Ras-dependent and Ras-independent pathways. Ligand activation leads to the phosphorylation of five tyrosine residues in the intracellular domain of NTRK1: Tyr490 in the juxtamembrane region, Tyr670, Tyr674, Tyr675 within the TK domain, and Tyr785 in the carboxyl-terminal tail. Phosphorylated Tyr490 provides the docking site for and mediate the phosphorylation and activation of Shc and SNT1/FRS2, two adaptor proteins signalling through the Ras/MAPK cascade; phosphorylated Y785 recruits and activates PLCγ (Kaplan and Miller, 1997; Meakin *et al*, 1999). The other three tyrosines are required for kinase activity, and recent studies have shown that they are also involved in the activation of rAPS and SH2-B (Qian *et al*, 1998) and the direct recruitment of Grb2 (Macdonald *et al*, 2000). The common downstream effectors of Shc, FRS2, Grb2, rAPS and SH-2B are ERK1/2 MAP kinases, followed by the activation of transcription factors and the induction of immediate early genes.

Shc adaptor proteins are cytoplasmic substrates for a number of receptor and non-receptor tyrosine kinases (TKs), including NTRK1 (van der Geer *et al*, 1995). By both alternative splicing and transcription start sites three protein isoforms of 66, 52 and 46 kDa are produced. The Shc p52/46 isoforms are involved in the transmission of signals from activated TKs (Migliaccio *et al*, 1997), and the Shc p66 isoform controls stress apoptotic responses and life span (Migliaccio *et al*, 1999). In addition to collagen homology (CH) domains, Shc proteins have phosphotyrosine binding (PTB) and Src homology 2 (SH2) domains (Migliaccio *et al*, 1997; Pelicci *et al*, 1992) both involved in exclusive or cooperative binding to phosphotyrosine residues. Upon receptor binding, Shc undergoes phosphorylation at three tyrosine residues (317, 239 and 240), becomes capable of interacting with the SH2 domain of Grb2, and causes the activation of the SOS/Ras/MAPK pathway. Different roles have been ascribed to Tyr317 and Tyr239/240, depending on the cell environment and on the kinase that trigger Shc activation. In hematopoietic cells and in EGF-stimulated NIH3T3 phosphorylation of Tyr317 has been proposed to trigger MAPK activation. On the contrary, phosphorylated Tyr239/240 signal to c-*myc* and thus regulate IL-3 dependent apoptosis in haematopoietic cells and contribute to the EGF-induced proliferation of NIH3T3 fibroblasts (Gotoh *et al*, 1996, 1997). In RAT-2 cells transformed by VEGF receptor, Tyr239/240 mediate an inhibitory signal for cell growth (Fournier *et al*, 1999). In PC12 cells Tyr239/240, but not Tyr317, play an important contribution to the NGF-induced Ras/MAP kinase activation and differentiation (Thomas and Bradshaw, 1997). It is therefore possible that the signalling pathway of Tyr239/240 may involve effectors other than Grb2.

The involvement of Shc in NTRK1 signal transduction has been demonstrated by various studies (Obermeier *et al*, 1993; Stephens *et al*, 1994). In a previous work we detected interaction of Shc SH2 domain with the wild type NTRK1 receptor and various TRK oncogenes expressed in NIH3T3 cells (Borrello *et al*, 1994). After the subsequent discovery of the Shc PTB domain, experiments with PC12 cells showed that Shc PTB domain interacts with Tyr490 of the activated NTRK1 receptor, whereas the interaction with an unidentified p115 phosphoprotein was proposed for the SH2 domain (Dikic *et al*, 1995).

As we are interested in elucidating the signal transduction pathways leading to the TRK oncogene transformation of NIH3T3 cells, we began by investigating the involvement of the proteins known to transduce NTRK1 signals. This apparently obvious approach is not as trivial as it may seem because NGF-induced NTRK1 activation leads to cell type specific biological effects, such as the differentiation of neuronal cells, the apoptosis of medulloblastoma cells and the mitogenesis of non-neuronal cells (Muragaki *et al*, 1997). This suggests that the critical elements of NTRK1 signalling may vary in cell lines from different tissues. In addition to differences due to cell specificity, TRK oncogenes may trigger signal transduction pathways other than the wild type receptor as a consequence of the properties contributed by structural rearrangements (cytoplasmic localization, the presence of activating sequences).

In a previous study we demonstrated that Shc is activated by TRK oncoproteins and therefore capable to recruit Grb2 (Borrello *et al*, 1994). We have more recently also determined the activation of FRS2, PLCγ, ERK1/2 and JNK MAP kinases in cells transformed by TRK-T3 (Greco *et al*, manuscript in preparation), as well as the recruitment of IRS1 and IRS2 by NTRK1 and TRK-T1 oncogene (Miranda *et al*, 2001).

In the study reported here we investigated the contribution of the Shc adaptor in TRK-T3 signal transduction. The mutation of the docking site for both Shc and FRS2 abolished the TRK-T3 biological activity, thus indicating that possible other pathways might not overcome the lack of the two adaptors. To further define the role of Shc in TRK-T3 activity we used the ShcY317F dominant-negative mutant, in which one of the tyrosines recruiting the Grb2/SOS complex has been mutated to phenylalanine. Our data show that ShcY317F reduces TRK-T3 biological activity, thus indicating the indispensability of Shc. Moreover, we report evidences that cells expressing both TRK-T3 and ShcY317F undergo cell death by a mechanism involving apoptosis.

## MATERIALS AND METHODS

### Plasmid construction

The T3/WT plasmid contains the TRK-T3 cDNA inserted into the pRC/CMV expression vector (Greco *et al*, 1995), which carries the neomycin resistance gene. The T3/Y291F mutant carries the mutation of Tyr291 to phenylalanine (F291) and was constructed by means of site-directed mutagenesis using an *in vitro* oligonucleotide mutagenesis system (Altered Sites *in vitro* Mutagenesis System, Promega). The T3/ABN kinase-defective mutant carries the mutation of the ATP binding site Lys339 to Ala (Greco *et al*, 1998). In ShcY317F mutant the Tyr317 is mutated to Phe. The cDNAs encoding ShcWT and ShcY317F are inserted in the pCGN expression vector, containing the hygromycin-resistance gene, and are tagged by the HA epitope at the N-terminus.

The VGF8-luc is a reporter plasmid made of *vgf* promoter sequence fused to the luciferase reporter gene (a kind gift from Dr R Possenti).

The pRL-CMV vector, containing the coding region of the Renilla luciferase (RL) gene, was provided by Promega.

### Cell culture and transfection

Mouse NIH3T3 fibroblasts were cultured in Dulbecco's modified Eagle's medium (DMEM) supplemented with 10% calf serum, human kidney 293T cells in DMEM supplemented with 10% foetal calf serum, and transformed cell lines in DMEM supplemented with 5% calf serum. PC12 cells were grown in RPMI-1640 medium supplementd with 5% foetal calf serum and 10% horse serum. NF797 cells are NIH3T3 cells transformed by the TRK-T3 oncogene; NWT and NY317F cell lines are derived from NIH3T3 cells respectively transfected with ShcWT and ShcY317F plasmids, and selected in the presence of hygromycin (25 μg ml^−1^). 3.9HG are semi-transformed cells generated by the transfection of NY317F cells with the TRK-T3 oncogene and isolated in medium supplemented with 5% calf serum plus hygromycin (25 μg ml^−1^) and G418 (400 μg ml^−1^).

The NIH3T3, NWT and NY317F cells (8×10^4^/60 mm plate) were transfected by the CaPO_4_ method as previously described (Bongarzone *et al*, 1989), using 250 ng of expression plasmid DNA together with 15 μg of mouse DNA. Transformed foci were selected in DMEM supplemented with 5% serum, in the presence or absence of hygromycin (25 μg ml^−1^), G418 (400 μg ml^−1^) or both. G418- and hygromycin-resistant colonies were selected in DMEM plus 10% serum containing the selective agents at the above concentrations. The transformed foci and resistant colonies were either fixed or isolated for further studies 2 weeks after transfection. The 293T cells were transiently cotransfected with T3/WT or T3/mutants (1 μg) together with Shc constructs or pCGN empty vector (5 μg) using the CaPO_4_ method. The cells were kept in 10% serum medium for 6–7 h, serum-starved overnight in DMEM containing 0.5% FCS, and then processed for protein extraction. PC12 cells were transfected using Cellfectin (Lifetechnologies, Inc.). Cells (2×10^5^) were seeded on collagen-coated 12-multiwell plates and transfected with 400 ng of T3/WT or T3/Y291F plasmid. In cotransfection experiments 100 ng of T3/WT together with 500 ng of ShcWT, ShcY317F or pCGN plasmid were used. Specific plasmid DNA were transfected together with 150 ng of VGF8-luc plasmid and 50 ng of pRL-CMV plasmid (Promega). Cells were incubated with the reagent for 7 h and scored for neurites outgrowth 2 days later.

### Luciferase activity assay

Both luciferase activities of VGF8-luc and pRL-CMV genes in PC12 cells lysates were measured using the Dual-luciferase reporter assay system (Promega). Assays were performed according to the manufacturer's recommendations. Light emission was measured using a TD-20/20 Luminometer.

### Microfocus forming assay

The assay was performed as described by Li *et al* (1999). One hundred cells from NF797 cell lines transfected with pCGN vector, ShcWT or ShcY317F were combined with 1×10^5^ NIH3T3 cells in 10-cm dishes, and cultured for 2 weeks in medium containing 5% calf serum. The foci were counted after GIEMSA staining.

### Immunoprecipitation, pull-down and Western blot analysis

Cells were lysed with PLCLB buffer (50 mM HEPES, 150 mM NaCl, 10% glycerol, 1% Triton X-100, 1.5 mM MgCl_2_, 1 mM EGTA, 10 mM Na_4_P_2_O_7_, 100 mM NaF) supplemented with aprotinin, pepstatin, leupeptin, PMSF and Na_3_VO_4_. One milligram of the cell extracts was precipitated with the appropriated antibodies, or with GST-Grb2(SH2) fusion protein conjugated to gluthatione-sepharose, or with p13suc1-agarose. The precipitates were washed three times with HNTG buffer (20 mM HEPES, 150 mM NaCl, 0.1% Triton X-100, 10% glycerol) and boiled in Laemmli sample buffer. Protein samples were electrophoresed on 8.5% SDS–PAGE, transferred onto nitrocellulose filters and immunoblotted with the appropriated antibodies. The immunoreactive bands were visualised using horseradish peroxidase-conjugated secondary antibody and enhanced chemiluminescence (Amersham). The anti-TRK and anti-Grb2 antibodies were from Santa Cruz Biothec., Inc.; the anti-HA antibodies were from BabCo; the anti-Shc, anti-FRS2, anti-phosphotyrosine antibodies and the p13suc1-agarose were from Upstate biothecnology, Inc.

### TUNEL analysis of DNA fragmentation

*In situ* detection of apoptotic cells was performed on adherent cells cultured on chamber slides by using the In Situ Cell Death Detection Kit, Fluorescein (Boehringer Mannheim), according to the manufacturer's instruction. The slides were then counterstained with 4′,6-diamin2-phenylindole.

## RESULTS

### TRK-T3 biological activity requires the Shc/FRS2 docking site

**Figure 1:**
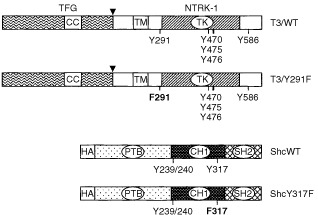
Schematic representation of TRK-T3 and Shc constructs. In the TRK-T3 constructs the portions contributed by TFG and NTRK1 are shown, with an arrowhead indicating the breakpoint. The coiled-coil (CC), transmembrane (TM) and tyrosine kinase (TK) domains are indicated. The tyrosine residues involved in Shc and FRS2 interaction (Y291), PLCγ interaction (Y586) and tyrosines of the activation loop (Y470, Y475 and Y476) are indicated. In T3/Y291F mutant the tyrosine 291 has been mutated to phenylalanine (F291). The TRK-T3 cDNAs were inserted into the pRC/CMV expression vector. The Shc constructs show the PTB domain, the collagen homology region (CH1) and the SH2 domain. Y239/240 and Y317 are tyrosine residues phosphorylated by tyrosine kinases. In ShcY317F, tyrosine 317 is mutated to phenylalanine (F317). The Shc cDNAs contain the HA epitope at the N-terminus and were inserted into the pCGN mammalian expression vector.

**Figure 2:**
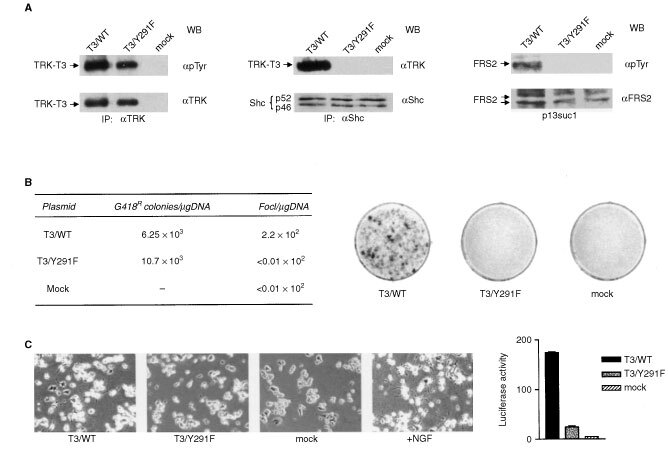
Biochemical and biological analysis of T3/Y291F mutant. (**A**) T3/WT or T3/Y291F expression plasmids were transiently transfected into 293T cells. Cell extracts were immunoprecipitated (IP) with the antibodies anti-TRK, anti-Shc or with p13suc1-agarose and blotted with anti-phosphotyrosine, anti-TRK, anti-Shc and anti-FRS2 antibodies. (**B**) Effect of the Y291F mutation on TRK-T3 transforming activity. NIH3T3 cells were transfected with the indicated constructs and subjected to G418 and foci selection. Plates were fixed and scored after GIEMSA staining 2 weeks later. (**C**) Effect of the Y291F mutation on TRK-T3 differentiating activity. PC12 cells were transfected with the indicated constructs and scored for the presence of neurites 3 days later. As control, untreated and NGF-stimulated (50 ng ml^−1^) PC12 cells are shown. The graph shows a quantification of differentiating activity. TRK-T3 constructs were cotransfected with VGF8-luc and Renilla plasmids; both luciferase activities were measured using the Dual-Luciferase reported assay system (Promega). The values were expressed relative to Renilla luciferase activity for normalization. The results presented are an average of three experiments.

The Tyr490 residue of the NTRK1 receptor is involved in the recruitment and activation of Shc adaptor proteins; such interaction occurs through the Shc PTB domain (Dikic *et al*, 1995). Tyr490 is also the docking site for SNT1/FRS2, a myristilated adaptor molecule signalling through Grb2/SOS/ERKs (Rabin *et al*, 1993; Kouhara *et al*, 1997; Ong *et al*, 2000). *In vitro* studies have shown a competition between Shc and FRS2 for the binding to the phosphorylated Tyr490 of NTRK1 (Meakin *et al*, 1999). With the final aim to provide evidences of possible oncogene-specific signal transduction pathways that could bypass the need for Shc and FRS2, we defined the role of TRK-T3 Tyr291 (corresponding to NTRK1 Tyr490) in the oncogene biological activity. The tyrosine residue was changed to phenylalanine by site-directed mutagenesis, to produce T3/Y291F (Figure 1

). Wild type and Y291F TRK-T3 cDNAs were inserted into the pRC/CMV mammalian expression vector, which also carries the neomycin resistance gene. Transient expression into human kidney 293T cells showed that, similarly to the wild type, the T3/Y291F mutant produced a phosphorylated 68 kDa protein (Figure 2A

). Western blot analysis of Shc immunocomplexes hybridised with anti-TRK (Figure 2A) showed that the T3/Y291F mutant is unable to interact with Shc (Figure 2A). The same cell extracts were incubated with the FRS2 interacting protein p13suc1 conjugated to agarose beads. The complexes immunoblotting with anti-FRS2 antibodies showed a constitutive association of FRS2 with p13suc1. However, immunoblotting with anti-phosphotyrosine antibodies showed that FRS2 is activated by TRK-T3 wild type but not by the T3/Y291F mutant.

We next analysed the transforming activity of T3/Y291F mutant by NIH3T3 transfection/focus formation experiments. As reported in Figure 2B, the transfection efficiency of T3/Y291F, determined as frequency of G418-resistant colonies, was slightly higher than wild type. However, the capacity to induce NIH3T3 transformation was completely abolished in the mutant oncogene. The biological effect of T3/Y291F mutant was also investigated in PC12 cells, in which the constitutively activated TRK oncogenes recapitulate the neuronal differentiating effect of NGF-stimulated NTRK1 receptor. As shown in Figure 2C T3/Y291F failed to induce neurites outgrowth. To quantify the differentiating activity of the TRK-T3 constructs we performed PC12 transfection in the presence of the VGF8-luc reporter plasmid; the latter is made of the luciferase reporter gene driven by the promoter of the *vgf*, a gene induced by NGF and considered a differentiation marker (Possenti *et al*, 1992; Canu *et al*, 1997). As shown by the graph in Figure 2C, the luciferase activity in cells expressing T3/Y291F is drastically reduced (86%) with respect to T3/WT.

All these results demonstrated that the Y291F mutation abrogates the TRK-T3 interaction with Shc and FRS2 adaptors and, consequently, its biological activity. This demonstrates that possible novel signal transduction pathways triggered by the rearranged versions of the NTRK1 receptor may not compensate the need of Shc and FRS2 signalling.

### Effect of ShcY317F mutant on TRK-T3-dependent signal transduction

As a tool for defining the role of Shc adaptor in TRK-T3 signal transduction we used the ShcY317F mutant of the 52 kDa Shc isoform (Figure 1), in which the tyrosine 317 has been changed to phenylalanine. Tyr317 of Shc, in addition to Tyr239/240, recruits Grb2 upon phosphorylation; the ShcY317F mutant has been shown to exert a dominant-negative effect in different biological processes mediated by Shc (Li *et al*, 1999; Stevenson *et al*, 1999; Boney *et al*, 2000; Mercalli *et al*, 2001). The ShcY317F and the control ShcWT constructs (Figure 1) were tagged by the haemoagglutinin (HA) epitope, which confers a small electrophoretic shift with respect to the endogenous p52 Shc, and cloned into the pCGN expression vector harbouring the resistance to hygromycin.

**Figure 3:**
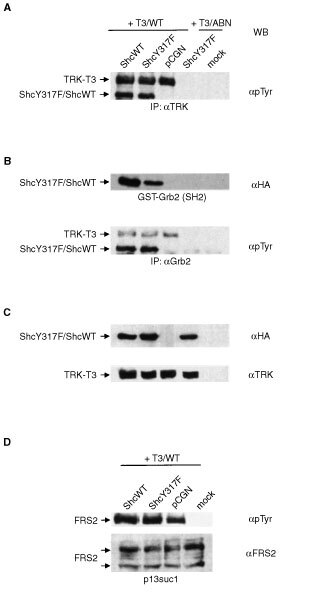
(**A**) ShcY317F is phosphorylated by TRK-T3. 293T cells were transiently cotransfected with the indicated constructs. Cell extracts were immunoprecipitated (IP) with anti-TRK and immunoblotted (WB) with anti-phosphotyrosine antibodies. The TRK-T3 oncoprotein, the 53 kDa HA-tagged ShcWT and ShcY317F transfected proteins are indicated. Cells cotransfected with T3/WT and pCGN vector were used as negative control. (**B**) Interaction of transfected Shc proteins with Grb2. The above described cell extracts were incubated with the GST-Grb2(SH2) fusion protein (top) or immunoprecipitated with anti-Grb2 antibodies (bottom) and hybridised with anti-HA and anti-phosphotyrosine antibodies, respectively. The phosphorylated proteins present in the anti-Grb2 immunocomplexes are indicated. (**C**) To show the expression of the transfected proteins, cell extracts above described were immunoblotted with anti-HA and anti-TRK antibodies. (**D**) Effect of ShcY317F mutant on TRK-T3 induced FRS2 activation. The same cell extracts above described, except T3/ABN-ShcY317F cotransfection, were pulled down with p13suc1-agarose and immunoblotted with anti-phosphotyrosine or anti-FRS2 antibodies.

We investigated the capability of ShcY317F to interact with TRK-T3 oncoprotein. Human kidney 293T cells were transiently cotransfected with TRK-T3 or T3/ABN kinase-dead mutant, together with HA-tagged ShcWT, ShcY317F or the pCGN vector as control (Figure 3

). The expression of the transfected proteins is shown in Figure 3C. Similarly to ShcWT, the 53 kDa phosphorylated ShcY317F protein coprecipitated with TRK-T3, as shown by the anti-phosphotyrosine Western blot hybridisation of anti-TRK immunocomplexes. This interaction required the phosphorylation of TRK-T3, as it was undetectable when the T3/ABN kinase-dead mutant (Greco *et al*, 1998) was used (Figure 3A).

We next investigated the ability of ShcY317F to recruit Grb2 upon TRK-T3 activation. The same cell extracts as above were incubated with the GST-Grb2(SH2) fusion protein, containing the Grb2 SH2 domain. Hybridisation with anti-HA antibodies (Figure 3B, top) showed that the amount of ShcY317F protein reacting with GST-Grb2(SH2) is lower than ShcWT, in accordance with the lack of an interaction site. The ShcY317F-Grb2 interaction was also investigated by coimmunoprecipitation experiments. The above cell extracts were immunoprecipitated with anti-Grb2 antibodies and hybridised with antiphosphotyrosine antibodies. Similarly to ShcWT and endogenous Shc proteins (data not shown), phosphorylated ShcY317F coprecipitated with Grb2 (Figure 3B, bottom). The ShcY317F-Grb2 interaction depends on the TRK-T3-triggered Shc activation, being undetectable when the T3/ABN kinase-dead mutant was used. Grb2 immunocomplexes also contain the phosphorylated TRK-T3 oncoprotein (Figure 3B, bottom), as consequence of both direct (Macdonald *et al*, 2000) and Shc/FRS2-mediated interaction. Altogether these results indicate that ShcY317F is phosphorylated by TRK-T3 at tyrosine residues 239/240 and therefore capable of recruiting Grb2.

As reported in the previous paragraph, both Shc and FRS2 bind, in a competitive manner, to phosphorylated NTRK1 Tyr490 residue (Meakin *et al*, 1999). We investigated the effect of ShcY317F on FRS2 activation induced by TRK-T3. The same cell extracts from serum-starved 293T cells transiently cotransfected with TRK-T3 and ShcWT, ShcY317F or the pCGN vector as control were pulled-down with the FRS2-interacting p13suc1 protein conjugated to agarose beads, and the eluted complexes analysed by Western blot. As shown in Figure 3D, hybridisation with anti-phosphotyrosine antibodies detected comparable levels of FRS2 phosphorylation in cells expressing TRK-T3 in combination with the pCGN empty vector, ShcWT or the ShcY317F mutant. As control, the expression of FRS2 is shown. These results indicate that, in our experimental conditions, although in the presence of exogenous Shc proteins, Tyr291 of TRK-T3 is still capable to signal through FRS2.

### Inhibitory effect of ShcY317F mutant on TRK-T3 biological activity

In the first paragraph we have shown that the biological activity of TRK-T3 is abrogated by the mutation of the Tyr291, docking site for Shc and FRS2. To directly explore the role of the Shc adaptor in TRK-T3 signal transduction, we investigated the effect of the ShcY317F dominant-negative mutant on TRK-T3 biological activity.

**Figure 4:**
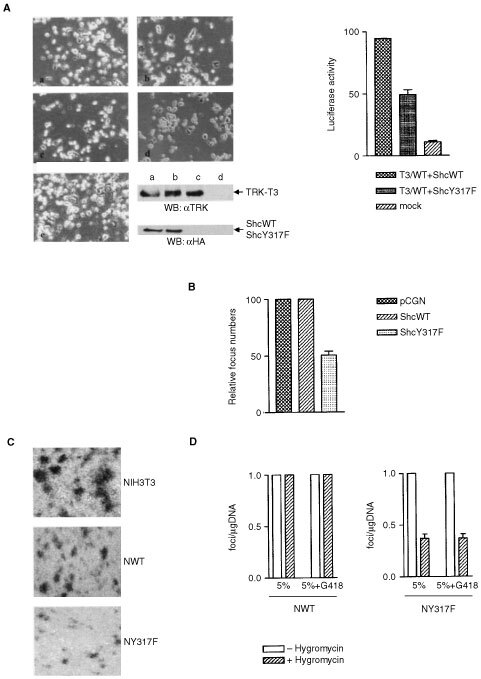
(**A**) Effect of ShcY317F on TRK-T3 induced PC12 differentiation. PC12 cells were cotransfected with TRK-T3 and ShcWT (a), ShcY317F (b) or pCGN (c) plasmids, together with VGF8-luc and Renilla plasmids, as described in Materials and Methods and scored for the presence of neurites 3 days later. As control, untreated (d) and NGF-stimulated (50 ng ml^−1^) (e) PC12 cells are shown. After morphological analysis, transfected PC12 cells were lysates and subjected to immunoblot using anti-TRK and anti-HA antibodies to detect the level of transfected proteins. To quantify the TRK-T3 differentiating activity both luciferase activities were measured using the Dual-Luciferase reported assay system (Promega). The values were expressed relative to Renilla luciferase activity for normalisation. The results presented are an average of three experiments. (**B**) Microfocus formation assay. One hundred cells from focus NF797 (NIH3T3 cells transformed by TRK-T3 oncogene) transfected with pCGN vector, ShcWT or ShcY317F constructs were combined with 1×10^5^ NIH3T3 cells in 10-cm dishes, and cultured for 2 weeks in medium containing 5% calf serum. The foci were counted after GIEMSA staining. (**C**) Transforming activity of TRK-T3 in NIH3T3, NWT (NIH3T3 cells stably expressing ShcWT) and NY317F (NIH3T3 cells stably expressing ShcY317F) cells. Transfection and foci selection were performed as described in Materials and Methods. Foci and G418 resistant colonies were either fixed and GIEMSA stained or isolated for further analysis after two weeks of selection. (**D**) Effect of hygromycin on TRK-T3 foci formation in NWT and NY317F cell lines. Foci selection was performed in 5% serum medium supplemented or not with 400 μg ml^−1^ of G418 (selectable marker contained in the TRK-T3 expression vector) in the absence or presence of 25 μg ml^−1^ hygromycin (selectable marker contained in the Shc expression vector). Foci were scored after 2 weeks of selection. For each cell line, the number of foci per μg DNA selected in the absence of hygromycin was set at 100%.

We first investigated the effect of ShcY317F on TRK-T3 differentiating activity. PC12 cells were cotransfected with TRK-T3 together with HA-tagged ShcWT, ShcY317F or the pCGN empty vector. The results are reported in Figure 4A

. Three days after transfection cell transfected with TRK-T3 and ShcWT or pCGN displayed long neurites. On the contrary, cells transfected with TRK-T3 and ShcY317F showed shorter extentions, with a few of them exceeding the cell diameter. As control the expression of TRK-T3 and HA-tagged Shc proteins is reported (Figure 4A). The differentiating activity was measured by cotransfecting the VGF8-luc reported plasmid, as above described. Luciferase activity in cells expressing T3/WT and ShcY317F was reduced to 50% with respect to cells expressing T3/WT and ShcWT (graph in Figure 4A).

We next investigated the effect of ShcY317F mutant on TRK-T3 transforming activity. As first approach we performed the microfocus formation assay. NF797 cells (NIH3T3 transformed by the TRK-T3 oncogene) were transfected with ShcY317F and ShcWT or the empty pCGN vector as control, as described in Materials and Methods. Transfected cells were mixed with an excess of wild type NIH3T3 cells, seeded in medium containing 5% calf serum, and their ability to form foci was assessed. As shown in Figure 4B focus forming capability was unaffected in the NF797 cells transfected with pCGN and ShcWT, but was reduced by about 50% in the cells expressing ShcY317F.

To more directly explore the effect of ShcY317F on TRK-T3 induced transformation we constructed NIH3T3 cell lines expressing exogenous ShcWT and ShcY317F proteins. The Shc constructs (Figure 1), as well as the pCGN empty vector, produced hygromycin-resistant NIH3T3 colonies with equivalent efficiency (data not shown), thus demonstrating that the overexpression of wild type and Y317F Shc proteins does not interfere with the growth of normal NIH3T3 cells. Several colonies were isolated and analysed for the expression of the transfected Shc constructs (data not shown). Selected cell lines (clone NWT for ShcWT and clone NY317F for ShcY317F) were used as recipients for focus formation assay. Both cell lines were transformed by Ha-ras and TRK-T3 oncogenes with comparable efficiency (data not shown). However, whereas the Ha-ras foci displayed similar size in both recipient cell lines (data not shown), the foci induced by TRK-T3 in NY317F cells were smaller than those in NIH3T3 and NWT (Figure 4C). This difference is related to the inhibitory effect of ShcY317F rather than the growth rate of the NY317F parental cell line, as deduced by growth curve determination (unpublished observations).

In order to characterise further the inhibitory effect of ShcY317F on TRK-T3 transforming activity, we investigated the influence of the level of Shc mutant protein expression. To this aim we performed foci selection in the presence of hygromycin, whose resistance is carried by the pCGN expression vector, based on previous observations showing that the level of ShcWT and ShcY317F proteins increases when NWT and NY317F cells are cultured in the presence of the antibiotic (unpublished results). As shown in Figure 4D, the addition of hygromycin did not affect the transforming activity of TRK-T3 in NWT cells. On the contrary, hygromycin reduced the number of foci induced by TRK-T3 in NY317F cells by about 60%, both in the presence and in the absence of G418, whose resistance is carried by the TRK-T3 expression vector. The presence of hygromycin also reduced the size of foci in NY317F cells (data not shown). These results support the view that ShcY317F exerts an inhibitory effect on TRK-T3 transforming activity, which can be modulated by the protein amount.

### Long-term effects of ShcY317F on the TRK-T3 induced transformed phenotype

Having shown that ShcY317F reduced TRK-T3 transforming activity, we were interested in determining any long-term effect in foci induced by TRK-T3 oncogene in NY317F cells, expressing the ShcY317F mutant. Several foci were isolated, in the presence or absence of hygromycin and/or G418 (as above reported, Figure 4D) with the aim of enhancing the expression of ShcY317F and/or TRK-T3 proteins. Interestingly, a difference was evident after isolation: the morphology of the TRK-T3 foci in NY317F cells was less transformed than that of the foci in NIH3T3 and NWT cells, and will be subsequently called ‘intermediate’ (Table 1

**Table 1:**
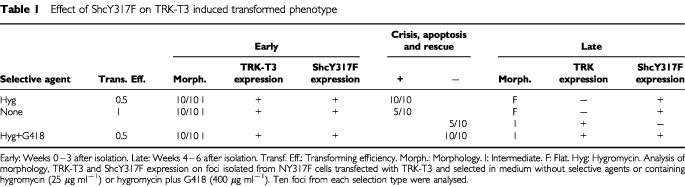
Effect of ShcY317F on TRK-T3 induced transformed phenotype

). Soon after isolation (‘early’ in Table 1), Western blot analysis and/or immunofluorescence revealed that all the foci in NY317F cells expressed both TRK-T3 and ShcY317F proteins (data not shown). Later on the behaviour of the foci during culture varied according to the method of selection. The foci selected in the presence of hygromycin, which increased the level of ShcY317F protein, underwent a growth crisis starting 4 weeks after isolation. Most of the cells died; the surviving cells had a flat phenotype, and they could be rescued and propagated by increasing the serum concentration up to 10% (‘late’ in Table 1). At this stage Western blot and/or immunofluorescence analysis of the rescued cells showed loss of TRK-T3 expression, but no changes in ShcY317F expression. The same effect was observed in half of the foci isolated in the absence of selective agents, whereas the remaining 50% maintained the intermediate phenotype and the expression of TRK-T3 protein, but lost the expression of ShcY317F. The foci selected in the presence of both hygromycin and G418 maintained the intermediate phenotype and the expression of both TRK-T3 and ShcY317F proteins. Altogether these results indicate that TRK-T3 full transforming activity requires signaling through tyrosine 317 of Shc.

To get inside the cell death described above, the TRK-T3 induced NY317F foci were monitored twice a week for the occurrence of apoptosis by TUNEL. Although there was considerable variability among the clones, 2–10% of TUNEL-positive cells was detected in those undergoing a growth crisis and loosing TRK-T3 expression. By contrast, when the parental NY317F cell line or a NIH3T3 focus transformed by TRK-T3 were analysed, no TUNEL-positive cells were scored. Furthermore, the number of TUNEL-positive cells may have been underestimated because TUNEL picks up the late stage of apoptosis marked by DNA breaks. These results suggest that the inhibitory effect of ShcY317F on TRK-T3 transforming activity involves apoptosis.

**Figure 5:**
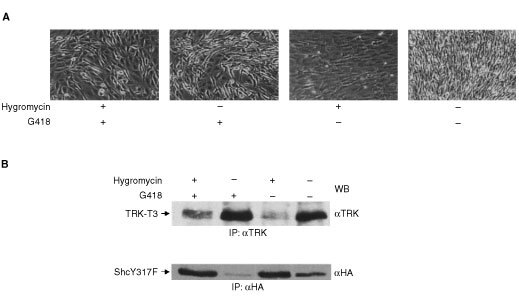
Effect of selective agents on the phenotype and TRK-T3 and ShcY317F protein expression on focus 3.9HG. (**A**) The 3.9HG cell line, generated by transfection of NY317F cells with TRK-T3, was selected in medium containing both hygromycin (25 μg ml^−1^) and G418 (400 μg ml^−1^) and displayed an ‘intermediate’ phenotype. The 3.9HG cells were cultured for four weeks in the absence or in the presence of different combinations of selective agents (hygromycin+G418, G418, hygromycin). (**B**) Cell extracts from the 3.9HG cells cultured under the four different conditions were immunoprecipitated and immunoblotted with anti-TRK and anti-HA antibodies to show the level of TRK-T3 and ShcY317F proteins.

The effect of selective agents on the transformed phenotype and ShcY317F and TRK-T3 protein expression was recapitulated on focus 3.9HG, which was selected and expanded in the presence of both hygromycin and G418, and had an ‘intermediate’ phenotype. The cells were cultured in the presence and absence of hygromycin and/or G418 (Figure 5

). After one month, the cells kept in the absence of selective agents or in the presence of G418 alone were more transformed than those kept in the presence of both agents, whereas those exposed to hygromycin alone were completely flat (Figure 5A). Western blot analysis demonstrated that the level of TRK-T3 protein was enhanced by G418 and that of ShcY317F protein was enhanced by hygromycin. Furthermore, the expressions of TRK-T3 and ShcY317F were inversely correlated, and the transformed and flat phenotypes respectively correlated with high expression of TRK-T3 and ShcY317F (Figure 5B). These results confirm the considerations deduced from the focus formation assay in NY317F cells, and indicate that TRK-T3 induced transformation is maintained only if endogenous Shc signalling is preserved.

## DISCUSSION

TRK oncogenes, associated with a consistent fraction of human papillary thyroid carcinoma, are generated by somatic rearrangements and display constitutive tyrosine kinase activity (Pierotti *et al*, 1996). The biological effects of rearranged TRK oncogenes recapitulate that of NGF-stimulated wild type NTRK1 receptor. In fact, similarly to the activated receptor counterpart, TRK oncogenes induce transformation of NIH3T3 mouse fibroblasts and differentiation of rat pheochromocytoma PC12 cells. Analysis of TRK oncogenes signal transduction, performed in NIH3T3 foci, have detected the involvement of several signal transducers recruited by the NGF-stimulated NTRK1 receptor, such as Shc, PLCγ, ERK1/2 and JNK MAP kinases (Borrello *et al*, 1994, manuscript in preparation).

With the aim to understand the mechanism leading to thyroid transformation, we explored the role of the Shc adaptor protein in the signal transduction triggered by TRK-T3 oncogene. The mutation of TRK-T3 tyrosine 291, corresponding to the tyrosine 490 of the NTRK1 receptor, abolished the interaction with both Shc and FRS2 adaptor proteins. This led to the abrogation of TRK-T3 induced differentiation and transformation, thus demonstrating that signalling through tyrosine 291 is essential for oncogene activity and it cannot be compensated by possible TRK oncogene-specific signal transduction pathways.

To assess more directly the role of Shc in TRK-T3 activity, we used the ShcY317F mutant, carrying the mutation of the tyrosine 317 which, in addition to tyrosines 239/240, is involved in Grb2 recruitment. The ShcY317F mutant has been shown to exert a dominant-negative effect on the endogenous Shc activity in different contexts. In ErbB2-positive breast cancer cell lines, ShcY317F blocks growth by disrupting cell-cycle progression (Stevenson *et al*, 1999); in NIH3T3 cells it reduces the RET/PTC2 oncogene transforming activity (Mercalli *et al*, 2001); in adipocytes it suppresses the IGF-induced proliferation (Boney *et al*, 2000); moreover, a Shc mutant lacking the CH1 domain that includes Y317, suppresses NIH3T3 transformation induced by the neu oncogene (Li *et al*, 1999).

The evidence that the ShcY317F mutant is tyrosyl phosphorylated when coexpressed with TRK-T3 indicates that the oncogene targets residues Tyr239/240 of Shc. However, the capabilty of phosphorylated ShcY317F to interact with Grb2 is reduced with respect to ShcWT. The expression of ShcY317F protein did not affect the capability of TRK-T3 to recruit and activate FRS2. Nevertheless, ShcY317F had an inhibitory effect on TRK-T3 induced differentiation and transformation, thus supporting the hypothesis that Shc signalling through Y317 is indispensable for TRK-T3 activity. Our data on NIH3T3 transformation showed that such inhibitory effect depends on the relative amount of TRK-T3 and ShcY317F proteins. Indeed, inhibition is more evident by adding hygromycin to the selection medium: this increases the amount of ShcY317F protein, thus producing the greatest competition with endogenous Shc for binding to TRK-T3. ShcY317F produced effect not only on the foci number, but also on their morphology, which was manifest upon isolation: foci arose in the presence of ShcY317F were less transformed, and were named ‘intermediate’. In addition to these short-term effects on transforming activity, a long-term effect was observed in the cell lines expressing both TRK-T3 and ShcY317F proteins selected as foci of ‘intermediate’ phenotype. In most cases the clones underwent cell death, and the surviving population lost the transformed morphology and TRK-T3 oncogene expression. Other clones maintained the transformed morphology and oncogene expression either because the ShcY317F protein was not sufficient to titer out the wild type or because they compensated the inhibitory effect of ShcY317F by accessory mechanisms. The inhibition of TRK-T3 activity by ShcY317F is therefore related to effect on both proliferation and transformation.

In clones undergoing cell death we observed a significant number of apoptotic cells. This finding is in keeping with recent data suggesting that Shc plays a role in promoting cell survival and counteracting apoptosis in response to cytokine receptors (Gu *et al*, 2000) and NGF receptor (Ulrich *et al*, 1998; Ashcroft *et al*, 1999) stimulation. The occurrence of apoptosis in our experimental system can be explained by several hypotheses: (1) Shc might transduce possible TRK-T3 anti-apoptotic signals; (2) signalling through tyrosine 317 of Shc could counteract a possible pro-apoptotic pathway induced by TRK-T3; (3) a pro-apoptotic signal might be the consequence of cell stress due to incorrectly transduced TRK-T3 signalling. Interestingly, results similar to ours have been obtained by co-expressing ShcY317F and the Ret/PTC2 oncogene (Mercalli *et al*, 2001), thus indicating the existence of a mechanism shared by other RTK oncogenes.

In conclusion, our data demonstrate that signalling through ShcY317 is essential for TRK-T3 transformation. This residue may cooperate with Tyr239/240 and/or other pathways in such a way that, when mutated, the signalling is not sufficient for specific endpoints; alternatively, Tyr317 may itself trigger specific pathways. Furthermore, the inhibitory effect of ShcY317F may be due to competition not only with endogenous Shc, but also with other adaptors docking the same TRK-T3 site.

The relevance of these studies is the possibility to block the transforming potential of TRK oncogenes by interfering with signalling through the Y317 residue of Shc. However, the mechanism responsible for this inhibitory effect, in particular the apoptotic process, deserves further investigations.
