# Biological activity of the thyroid TRK-T3 oncogene requires signalling through Shc

**DOI:** 10.1038/bjc.2016.131

**Published:** 2016-05-26

**Authors:** E Roccato, C Miranda, V Ranzi, M Gishizki, M A Pierotti, A Greco

**Correction to:** *British Journal of Cancer* (2002) **87**, 645–653; doi:10.1038/sj.bjc.6600544; published online 3 September 2002

The corresponding author of the above paper, Dr Angela Greco, has informed us that due to an inadvertent and minor error in the preparation of the figure, the images labelled ‘T3/Y291F’ and ‘Mock’ in Figure 2B are probably duplicates. The images are photographs of tissue culture plates related to a focus formation assay in NIH3T3 cells in which both the samples, ‘T3/Y291F’ and ‘Mock’, were scored negative. The results of the assay are also reported in the Table in Figure 2B. Although the author regrets this error, it in no way affects the validity of the results and conclusions of the paper, which are also supported by the other experiments.

